# Monthly Record of Deaths in the City of Buffalo

**Published:** 1858-01

**Authors:** C. L. Dayton

**Affiliations:** Health Physician


ART. VII.— *Report of Mortality in Buffalo for the month of Nov.*, 1857.
By C. L. DAYTON, M. D., Health Physician.

| DISEASES. | No. | Males. | Females. |
|---|---|---|---|
| Accident, | 1 | 1 | |
| Asphyxia Immersorum, | 2 | 1 | 1 |
| Apoplexia, | 1 | | 1 |
| Cholera Infantum, | 5 | 2 | 2 |
| Croup, | 3 | 2 | 1 |
| Diarrhœa, | 3 | 1 | 2 |
| Dysentery, | 3 | 2 | 1 |
| Dentition, | 2 | 1 | 1 |
| Debilitas Senilis, | 6 | 2 | 4 |
| Delirium Tremens, | 1 | 1 | |
| Eclampsia, | 10 | 4 | 5 |
| Erysipelas, | 1 | 1 | |
| Enteritis, | 1 | | 1 |
| Febris Typhoid, | 5 | 3 | 2 |
| " Typhus, | 1 | | 1 |
| " Scarlatina, | 4 | 1 | 3 |
| Gastritis Acuta, | 1 | 1 | |
| Hyperæmia Cerebralis, | 2 | | 1 |
| " Pulmonalis, | 2 | 1 | 1 |
| Hydrops, | 5 | 3 | 2 |
| Hæmorrhage, | 1 | | 1 |
| Hydrocephalus, | 2 | 1 | 1 |
| Ignotus, | 22 | 11 | 8 |
| Intemperance, | 3 | 1 | 2 |
| Marasmus, | 3 | 2 | 1 |
| Morbus Cordis, | 3 | 3 | |
| Peritonitis, | 1 | | 1 |
| Pneumonitis, | 11 | 6 | 3 |
| Pertussis, | 2 | | 1 |
| Rubeola, | 3 | 3 | |
| Still-born, | 5 | 3 | |
| Tuberculosis Pulmonalis, | 12 | 5 | 7 |
| Total, | 127 | | |

SEXES.

| | |
|---|---|
| Males, | 62 |
| Females, | 54 |
| Sex not given, | 11 |
| Total, | 127 |

AGES.

| | | | |
|---|---|---|---|
| Still-born, | 5 | Between 20 years and 30 years, | 11 |
| 1 day, | 3 | " 30 " " 40 " | 9 |
| 1 day and 30 days, | 15 | " 40 " " 50 " | 7 |
| Between 1 month and 6 months, | 15 | " 50 " " 60 " | 1 |
| " 6 months and 12 " | 13 | " 60 " " 70 " | 5 |
| " 1 year " 3 years, | 21 | " 70 " " 80 " | 3 |
| " 3 " " 5 " | 7 | " 80 " " 90 " | 1 |
| " 5 " " 10 " | 5 | " 90 " " 100 " | 1 |
| " 10 " " 20 " | 6 | " 100 " | 0 |
| Total number under 10 years, | 83 | Total number over 10 years, | 44 |
| Ages not given, | 0 | Total, | 127 |

NATIVITIES.

| | | | |
|---|---|---|---|
| American, (white,) | 72 | Prussian, | 0 |
| " (colored,) | 3 | Swiss, | 1 |
| German, | 18 | French, | 2 |
| Irish, | 14 | Scotch, | 2 |
| English, | 3 | Poland, | 0 |
| Canadian, | 0 | Country not given, | 12 |
| Total, | | | 127 |

---

*Detection by Etherialization of Feigned Idiotcy.*—Etherization has been resorted to in Belgium as a means of acquiring judicial information. After a considerable robbery committed at Brussels, in November last, two men, named Lorch and Daubner, were arrested and brought to trial. The former was condemned to hard labor for life; but in consequence of the latter pretending to be dumb and idiotic, his trial was postponed, in order that a medical investigation should take place. It was found impossible to get even a sign of intelligence from him. As it was, however, known that he was not born dumb, and that he had spoken, when he said that he could speak no language but German, he was etherized, and while laboring under the effect of that application, he spoke perfectly, and in French. He was in consequence again brought before the tribunal, and condemned to ten years' hard labor.—*British Med. Journal*, Aug. 1st, 1857.

